# Resistance to thrips (*Enneothrips flavens)* in wild and amphidiploid *Arachis* species

**DOI:** 10.1371/journal.pone.0176811

**Published:** 2017-05-04

**Authors:** Marcos Doniseti Michelotto, Ignácio José de Godoy, Melina Zacarelli Pirotta, João Francisco dos Santos, Everton Luiz Finoto, Alessandra Pereira Fávero

**Affiliations:** 1 São Paulo Agency for Agribusiness Technology (APTA), Polo Centro Norte, Pindorama, São Paulo, Brazil; 2 Agronomic Institute (IAC), Campinas, São Paulo, Brazil; 3 Faculdade de Ciências Agrárias e Veterinárias, Universidade Estadual Paulista “Júlio de Mesquita Filho” (FCAV/Unesp), Jaboticabal, São Paulo, Brasil; 4 Embrapa Pecuária Sudeste, São Carlos, São Paulo, Brazil; US Department of Agriculture, UNITED STATES

## Abstract

*Thrips (Enneothrips flavens*) is a pest that causes severe damage and yield losses to peanut crop if not properly controlled. The main control method currently used by farmers is bi-weekly application of insecticides during crop development, which, in addition to its toxicity, is very costly. Thus, new sources of resistance must be identified in order to reduce the use of insecticides and effectively manage the pest. This study aimed to evaluate the occurrence and symptoms of *E*. *flavens* infestations in 12 accessions of 10 wild species of *Arachis* and nine amphidiploids, as well as to compare their morphoagronomic characteristics to those of commercial cultivars. To this end, we conducted experiments during two summer seasons, using a randomized block design with four replications. We conducted evaluations of the severity of infestation, noting visual symptoms of *E*. *flavens* and morphological and reproductive characteristics of the *Arachis* plants. Results indicated that wild accessions V 7635 (*A*. *vallsii*), V 13250 (*A*. *kempff-mercadoi*), K 9484 (*A*. *batizocoi*), Wi 1118 (*A*. *williamsii*), V 14167 (*A*. *duranensis*) and V 13751 (*A*. *magna*) are the most promising for obtaining useful new amphidiploids. Among the amphidiploids, An 12 (*A*. *batizocoi x A*. *kempff-mercadoi*)^4x^, An 9 (*A*. *gregoryi x A*. *stenosperma*) ^4x^, and An 8 (*A*. *magna x A*. *cardenasii*)^4x^ showed high level of resistance to *E*. *flavens*. The identified thrips resistant wild and amphidiploid *Arachis* species may be used in future breeding program to produce thrips resistant peanut cultivars.

## Introduction

The thrips *Enneothrips flavens* Moulton (Thysanoptera: Thripidae) is considered a major pest of peanut due to its widespread occurrence and high population levels [1]. The main control method currently used by farmers is bi-weekly application of insecticides during crop development. During infestations, *E*. *flavens* lodges within buds and developing shoots, damaging the vegetative growth of plants. Because of its mode of attack, the chemical products required for its control have compositions and rates of application that make them costly.

The use of cultivars with resistance to *E*. *flavens* could lead to gains in productivity or could promote a significant reduction in production costs by reducing or eliminating chemical control [2]. Thus, the search for resistant varieties is very important for the genetic improvement of the species. In Brazil, studies searching for resistance have been conducted with genotypes of the cultivated peanut, *Arachis hypogaea* L. [3–7], and the form of resistance known as "tolerance" was observed in some cultivars. However, the degree of resistance seen in many cultivars is considered too small in order to reduce or eliminate chemical control.

An alternative approach is the incorporation into breeding programs of wild *Arachis* species that present great genetic variability and are a potential source of genes for important agronomic traits such as resistance to diseases and pests [8–13]. However, the potential of this variability in wild species of *Arachis* has not been widely studied. The main obstacle to such studies is that the vast majority of wild species are diploid, while the cultivated species (*A*. *hypogaea*) is allotetraploid. The barrier of ploidy, as well as other factors such as genomic differences between species, precludes hybridization between wild species and *A*. *hypogaea* [14]. Obtaining fertile amphidiploid individuals by using colchicine to double chromosomes is a viable route for the introgression of wild *Arachis* spp. genes into *A*. *hypogaea*, as it is possible to produce amphidiploids with the same or similar genomic constitution as the cultivated species [15].

To effectively identify resistant plant material, assessments done under field conditions in locations near commercial plantations are required in order to select accessions and amphidiploids that could be crossed with *A*. *hypogaea*. Therefore, this study aimed to evaluate the occurrence and symptoms of *E*. *flavens* infestations in the wild and amphidiploid species of *Arachis* as well as to compare their morpho-agronomic characteristics to those of commercial cultivars.

## Materials and methods

The experiments were performed during two summer seasons, 2011/2012 and 2012/2013, in an experimental area of the Agência Paulista de Tecnologia dos Agronegócios (APTA; São Paulo Agency for Agribusiness Technology), Polo Centro Norte, Pindorama, São Paulo, Brazil.

In the first season (2011/2012), the experiment consisted of 24 treatments (genotypes), comprising 12 accessions of 10 wild species of *Arachis*, nine amphidiploids, and three genotypes of *A*. *hypogaea* (including two commercial cultivars used as controls; Table 1). In the second season, two amphidiploids were not evaluated due to an insufficient number of seeds.

**Table 1:** Genotypes used in the study during the 2011/2012 and 2012/2013 seasons.

Genotypes		Name of *Arachis* species
An 2 -	V 6389 x V 9401	(*A*. *gregoryi x A*. *linearifolia*)^4x^
An 4 -	KG 30076 x V 14167	(*A*. *ipäensis x A*. *duranensis*) ^4x^
An 6 -	K 9484 x GKP 10017	(*A*. *batizocoi x A*. *cardenasii*) ^4x^
An 7 -	V 7635 x Wi 1118	(*A*. *vallsii x A*. *williamsii*) ^4x^
An 8 -	V 13751 x GKP 10017	(*A*. *magna x A*. *cardenasii*) ^4x^
An 9 -	V 6389 x V 12488	(*A*. *gregoryi x A*. *stenosperma*) ^4x^
An 10^a^ -	KG 30097 x V 15076	(*A*. *magna x A*. *stenosperma*) ^4x^
An 11 -	V 7635 x V 10229	(*A*. *vallsii x A*. *stenosperma*) ^4x^
An 12 ^a^ -	K 9484 x V 13250	(*A*. *batizocoi x A*. *kempff- mercadoi*) ^4x^
Parents/ Acessions	V 15076	*A*. *stenosperma*
	V 6389	*A*. *gregoryi*
	GKP 10017	*A*. *cardenasii*
	V 13751	*A*. *magna*
	Wi 1118	*A*. *williamsii*
	V 7635	*A*. *vallsii*
	K 9484	*A*. *batizocoi*
	V 10229	*A*. *stenosperma*
	KG 30097	*A*. *magna*
	V 14167	*A*. *duranensis*
	KG 30076	*A*. *ipaënsis*
	V 13250	*A*. *kempff- mercadoi*
Controls	IAC Caiapó	*A*. *hypogaea*
	V 12549	*A*. *hypogaea*
	IAC 503	*A*. *hypogaea*

^a^ Not evaluated during the 2012/2013 season

The seeds used in the experiments were pretreated with the commercial products Ethrel^®^ (active ingredient: ethephon) at a dose of 2.0 mL/kg seed in order to break seed dormancy, and the fungicide Vitavax^®^-Thiram 200 SC (carboxin + thiram) at a dose of 2.5 mL/kg seed to protect against soil fungi. Seeds were germinated in 200-mL plastic cups containing soil substrate and manure (3:1) and placed in the greenhouse. When plants reached a height of approximately 15 cm, they were transferred to the field.

In both trials, we used a randomized block with four replications. Each plot consisted of a row containing five plants spaced one meter apart, with 1.80 meters between rows.

Plants were sprayed every 15 days with the fungicide chloratalonil (Bravonil 500^®^) at a dose of 1.75 L ha^-1^ per application, alone or in an admixture with triazoles (Score^®^) at 0.2 L ha^-1^ or with strobilurins (Opera^®^) at 0.6 L ha^-1^, to prevent diseases such as late leafspot, *Cercosporidium personatum* (Berk. & Curtis Deighton), early leafspot, *Cercospora arachidicola* (Horii), web blotch, *Phoma arachidicola* (Marasas, Pauer & Boerema), peanut scab, *Sphaceloma arachidis* (Bit. & Jenk), rust, *Puccinia arachidis* (Speg), and *Rhizoctonia solani* (Kühn).

Weeds were controlled with pre-plant-incorporated herbicide application of trifluralin (Trifluralin Nortox^®^) at a dose of 2.5 L ha^-1^. Manual hoeing was performed whenever necessary during plant development.

### Infestation and visual symptoms of E. flavens

During the experiments, we conducted seven (between 35 to 95 DAP) and eight (between 45 to 110 DAP) evaluations in the 2011/2012 and 2012/2013 seasons, respectively, to determine the number of insects (adults and nymphs) in young leaflets that were still closed, examining 10 leaves per plot.

We also evaluated the symptoms of thrips infestation by assigning visual symptom scores to 10 leaflets on a scale from 1 (leaf with no symptoms) to 5 (completely infested leaves), based on previous scales [16, 17].

After graphical analysis, we selected evaluations that showed the highest number of insects per leaflet or the highest visual symptom scores and performed an analysis using both the selected evaluations and the mean of all evaluations conducted during the growing season for number of insects and symptom score. Scores were transformed into (x + 0.5)^1/2^, submitted to analysis of variance by F test, and means were compared by Tukey’s test at 5% probability.

In order to rank the genotypes by resistance, we created an attack index (AI) by multiplying the average number of *E*. *flavens* by the average symptom score. More resistant genotypes had lower AI values.

We conducted principal component analysis using SAS^®^ [18] and Microsoft Excel. The variables analyzed included the number of thrips per 10 leaflets, the visual symptom score, and the attack index, using the mean values from both the seasons.

### Morphological and reproductive traits

In the 2011/2012 season, plants were subjected to morphological and reproductive evaluations, namely: (1) height of the main stem (MSH, cm); (2) average branch length (ABL, cm); (3) average number of branch nodes (ABN); (4) length between nodes (LBN, cm); (5) number of seeds per plant (NSP); and (6) mean seed weight (MSW, mg). In the 2012/2013 season, we evaluated only the amphidiploids. However, we did not evaluate LBN but did evaluate the number of primary branches (NPB).

The values were submitted to analysis of variance by F test, and means were compared with the Scott-Knott test at 5% error probability.

## Results

### Infestation and visual symptom scores of E. flavens

**Fig 1:**
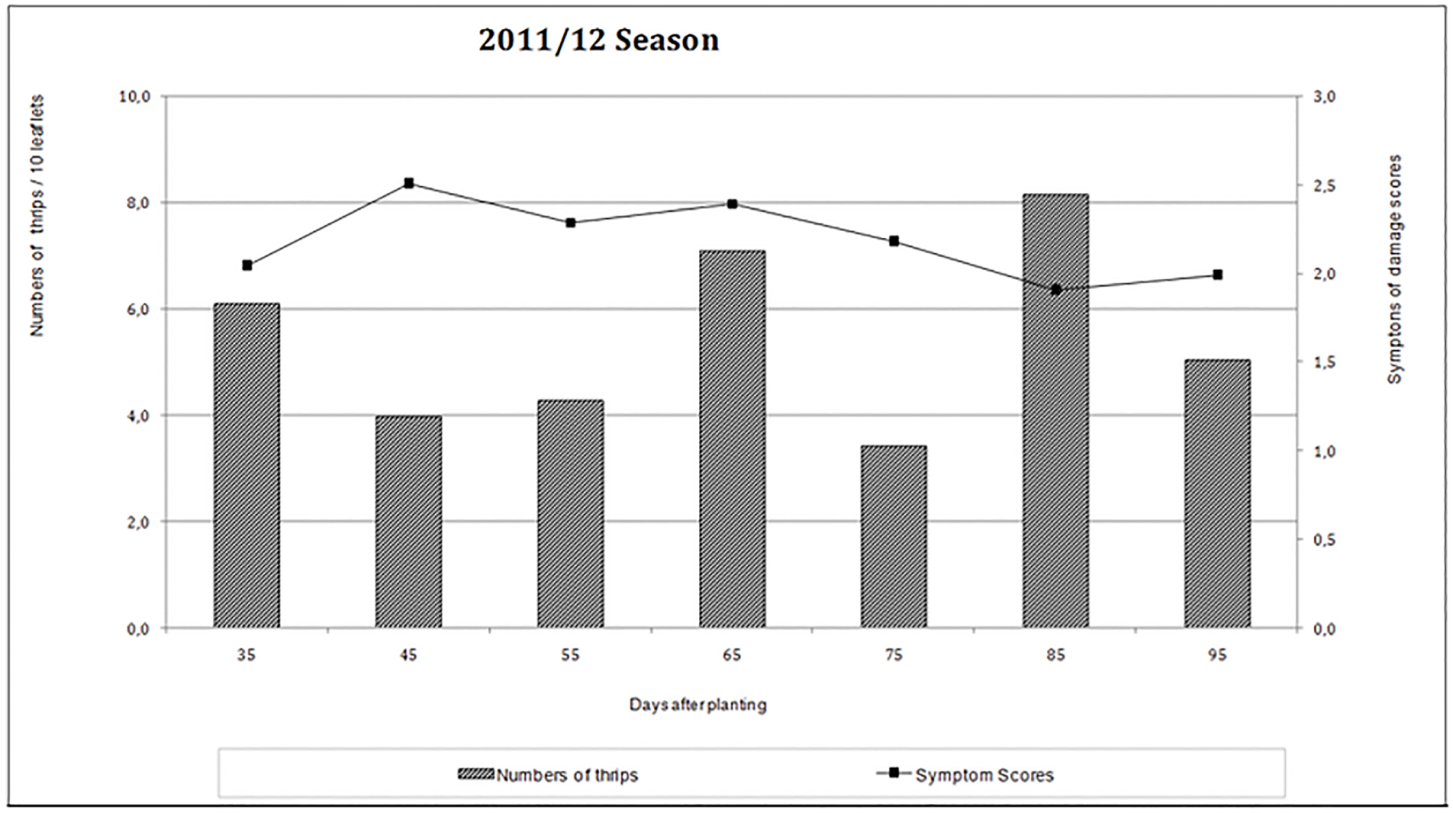
Number of thrips and visual symptom scores for infestation of peanut leaflets by *E*. *flavens* during the 2011/2012 growing season.

**Fig 2:**
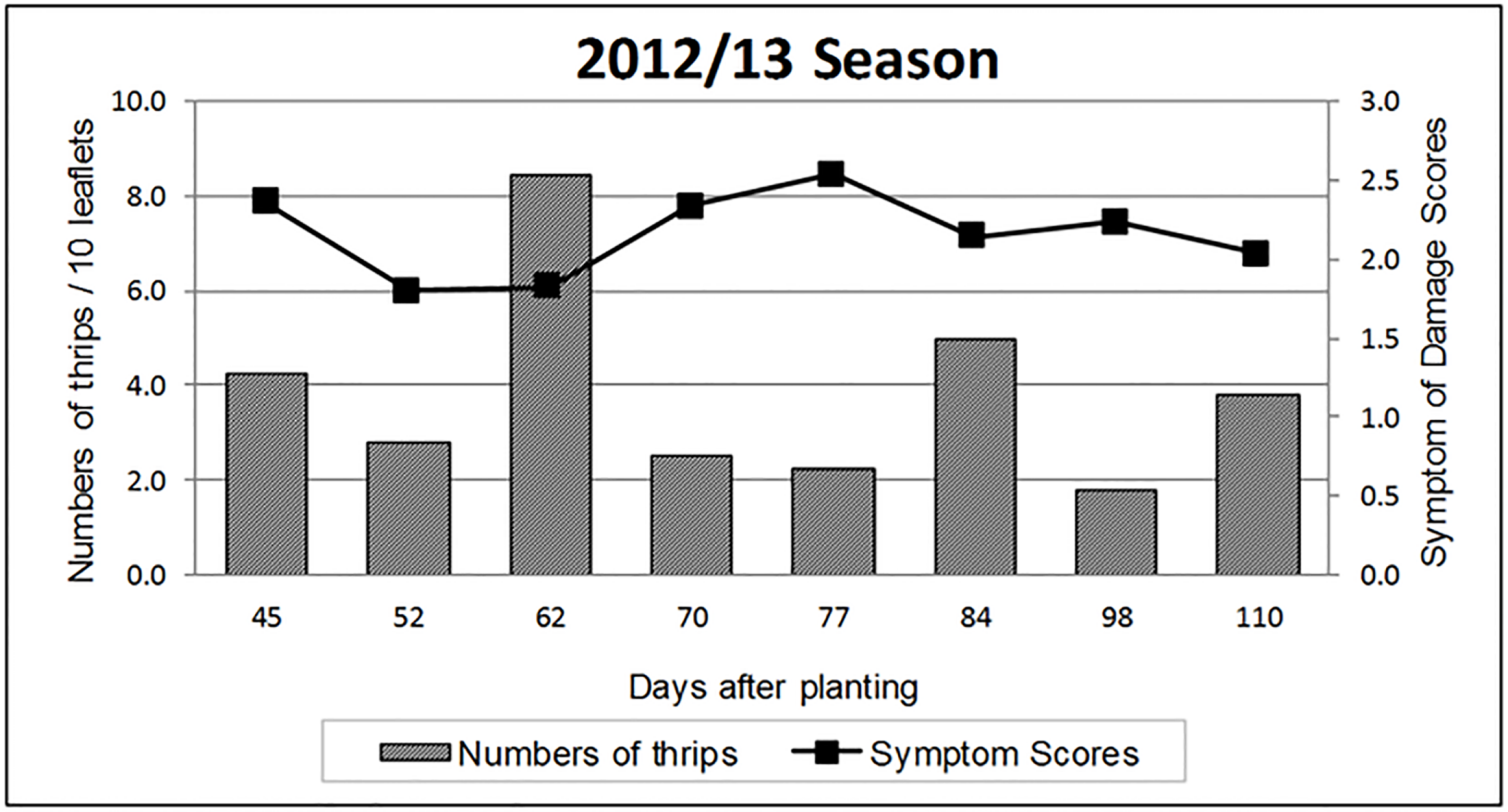
Number of thrips and visual symptom scores for infestation of peanut leaflets by *E*. *flavens* during the 2012/2013 growing season.

In both years, the insect population fluctuated unevenly over the course of the experiments, with some peaks of higher incidence (Figs 1 and 2). Visual symptom scores showed similar variation. In the 2011/2012 growing season, the level of thrips infestation was highest 85 days after planting (DAP), when a mean of approximately 8.0 thrips/10 leaflets was observed (Fig 1). The highest visual symptom score (2.5) was recorded 45 DAP.

In the 2012/2013 growing season, the highest number of thrips was observed 62 DAP, which had a mean similar to that of the previous year (8.0 thrips/10 leaflets; Fig 2). The highest visual symptom score occurred 77 DAP, with a mean score that was also similar to that of the previous season (2.5). The observed differences between years may be attributable to the effect of weather conditions on the field experiments.

When averaged over the seven evaluations conducted during the 2011/2012 growing season, the highest levels of infestation (> 10 thrips/10 leaflets) were observed in the control genotypes, with accession V 12549 showing the greatest number of thrips (11.7 thrips/10 leaflets) and highest AI (32.64; Table 2). The amphidiploids tested had intermediate levels of infestation, with the number of thrips per 10 leaflets ranging from 3.0 in An 12 (*A*. *batizocoi* x *A*. *kempff-mercadoi*)^4x^ to 7.1 in An 7 (*A*. *vallsii* x *A*. *williamsii*)^4x^. The lowest AI values were seen in the amphidiploids An 12 (*A*. *batizocoi* x *A*. *kempff-mercadoi*)^4x^ and An 8 (*A*. *magna x A*. *cardenasii*) ^4x^, which had AI values of 4.33 and 6.94, respectively (Table 2). The number of thrips per 10 leaflets at 85DAP had a high CV because the quantitative behavior of this characteristic.

**Table 2:** Number of thrips at 85 DAP and the mean of all seven evaluations, and visual symptom score at 45 DAP and the mean of all seven evaluations for the tested peanut amphidiploids, parents, and cultivars at Pindorama during 2011/2012 summer season.

Genotypes		Number/10 leaflets*		Symptom score*		Attack Index (AI)^a^
		85 DAP	Mean^a^	45 DAP	Mean^a^	
An 12 -	(K 9484 x V 13250)^4x^	8.7 abc	3.0 c-f	1.50 g	1.42 fg	4.33
An 8 -	(V 13751 x GKP 10017)^4x^	4.0 abc	3.4 c-f	2.50 a-g	2.04 c-f	6.94
An 11 -	(V 7635 x V 10229)^4x^	7.0 abc	4.9 b-f	2.23 b-g	2.01 c-f	9.85
An 2 -	(V 6389 x V 9401)^4x^	9.5 abc	5.2 b-f	2.31 a-g	1.95 c-g	10.14
An 6 -	(K 9484 x GKP 10017)^4x^	4.5 abc	4.2 b-f	3.26 a-d	2.50 a-d	10.50
An 7 -	(V 7635 x Wi 1118)^4x^	18.0 ab	7.1 a-d	1.77 efg	1.92 d-g	13.63
An 10 -	(KG 30097 x V 15076)^4x^	2.5 bc	5.6 b-f	2.86 a-g	2.45 a-e	13.72
An 9 -	(V 6389 x V 12488)^4x^	3.5 abc	5.9 a-f	3.17 a-d	2.55 a-d	15.05
An 4 -	(KG 30076 x V 14167)^4x^	4.0 abc	5.9 a-f	3.44 ab	2.83 ab	16.70
Parents	V 7635	2.5 bc	1.6 f	1.54 g	1.24 g	1.98
	V 13250	3.0 bc	2.1 ef	1.50 g	1.34 fg	2.81
	GKP 10017	3.9 abc	2.3 def	1.88 d-g	1.55 fg	3.57
	V 13751	5.5 abc	4.4 c-f	1.61 fg	1.49 fg	6.56
	K 9484	6.0 abc	3.6 c-f	2.03 c-g	1.89 d-g	6.80
	Wi 1118	6.5 abc	4.1 c-f	2.10 b-g	1.89 d-g	7.75
	KG 30076	2.0 c	5.5 b-f	2.05 c-g	1.93 c-g	10.62
	KG 30097	4.5 abc	5.6 b-f	2.10 b-g	1.94 c-g	10.86
	V 10229	5.0 abc	4.9 b-f	3.34 abc	2.60 a-d	12.74
	V 14167	4.0 abc	4.5 c-f	3.29 abc	3.00 ab	13.50
	V 15076	8.5 abc	6.6 a-e	3.13 a-e	2.35 b-e	15.51
	V 6389	12.5 abc	7.6 abc	3.69 a	3.16 a	24.02
Controls	IAC 503	24.4 a	10.9 ab	2.95 a-f	2.66 abc	28.99
	IAC Caiapó	14.0 abc	10.0 ab	3.08 a-e	3.01 ab	30.10
	V 12549	15.9 abc	11.7 a	2.69 a-g	2.79 ab	32.64
Mean		7.5	5.5	2.50	2.16	
F test		2.67**	6.65**	7.38**	17.02**	
CV (%)		40.63	9.24	20.56	12.78	

* Means followed by the same letter in a column did not differ significantly from each other according to Tukey’s test at 5% error probability.

^a^ Means of seven evaluations conducted during plant development. AI = mean number of thrips × mean symptom score.

** Significant at 1% error probability to F’s test.

The lowest levels of thrips infestation and lowest AI values observed during the 2011/2012 experiment were among the parental lines. Accession *A*. *vallsii* (V 7635) had the lowest values of the two measures (1.6 thrips/10 leaflets and AI 1.98). The highest level of infestation among the parents was found in *Arachis gregoryi* (V 6389), which had 7.6 thrips/10 leaflets and an AI of 24.02, surpassing the levels observed for the amphidiploids, which is in line with the great genetic variability found in wild species of the genus *Arachis* (Table 2, S1 Table). Relative to their parents, amphidiploids had AI values that were either intermediate, close to that of one parent, or higher than that of both parents. No amphidiploid showed resistance greater than both parents.

Generally, the number of thrips observed was positively correlated with the amount of damage to the plant. However, this was not true for the parents *A*. *gregoryi* (V 6389) and *A*. *duranensis* (V 14167), which had high visual symptom scores despite intermediate thrips values (Table 2).

In the second year of evaluation, the amphidiploids An 7 (*A*. *vallsii x A*. *williamsii*)^4x^, An 8 (*A*. *magna x A*. *cardenasii*)^4x^, and An 9 (*A*. *gregoryi x A*. *stenosperma*)^4x^ and the wild species accessions K 9484 (*A*. *batizocoi*), V 7635 (*A*. *vallsii*), and V 13250 (*A*. *kempff-mercadoi*) had the lowest levels of thrips infestation (Table 3). As in the previous year, presence of the pest was generally well correlated with plant damage as measured by the visual symptom score, although again there were exceptions. The amphidiploid An 7 (*A*. *vallsii x A*. *williamsii*)^4x^, for example, had one of the lowest pest levels but suffered relatively high damage. Similarly, the IAC Caiapó cultivar had low pest incidence but a high visual symptom score (Table 3). The IAC Caiapó cultivar is resistant to thrips, as its productivity is less affected by thrips attack in comparison to other cultivars [4].

**Table 3:** Number of thrips at 62 DAP and the mean of all eight evaluations, and visual symptom score at 77 DAP and the mean of all eight evaluations for the tested peanut amphidiploids, parents, and cultivars at Pindorama during 2012/2013 summer season.

Genotypes		Number/10 leaflets*		Symptom score*		Attack Index (AI)^a^
		62 DAP	Mean^a^	77 DAP	Mean^a^	
An 8 -	(V 13751 x GKP 10017)^4x^	3.5 cd	2.0 cd	1.65 ef	1.63 e-h	3.26
An 9 -	(V 6389 x V 12488)^4x^	3.5 cd	2.0 cd	2.00 def	1.70 e-h	3.40
An 11 -	(V 7635 x V 10229)^4x^	7.0 cd	2.5 cd	2.35 cde	2.10 cde	5.25
An 6 -	(K 9484 x GKP 10017)^4x^	6.0 cd	3.3 bcd	1.60 def	1.93 d-g	6.37
An 7 -	(V 7635 x Wi 1118)^4x^	3.5 cd	3.4 bcd	2.30 c-f	1.91 d-g	6.49
An 2 -	(V 6389 x V 9401)^4x^	8.0 bcd	4.5 bcd	2.35 cde	1.88 e-h	8.46
An 4 -	(KG 30076 x V 14167)^4x^	10.5 bc	5.5 bc	3.40 abc	2.55 bc	14.03
Parents/ Acessions	V 7635	2.0 cd	1.0 d	1.25 ef	1.53 fgh	1.53
	GKP 10017	5.0 cd	1.3 d	1.15 f	1.34 h	1.74
	V 13250	3.0 cd	1.8 cd	1.55 def	1.44 gh	2.59
	K 9484	0.5 d	1.5 cd	1.13 ef	1.99 def	2.99
	Wi 1118	4.0 cd	2.1 cd	1.75 def	1.64 e-h	3.44
	V 14167	5.0 cd	1.8 cd	1.85 def	1.93 d-g	3.47
	V 13751	8.5 bcd	2.0 cd	2.00 def	1.83 e-h	3.66
	KG 30097	5.5 cd	2.6 cd	2.05 def	1.81 e-h	4.71
	V 10229	7.5 bcd	2.8 cd	1.55 def	1.83 e-h	5.12
	V 15076	5.0 cd	2.9 cd	2.30 c-f	1.99 def	5.77
	V 6389	6.0 cd	2.9 cd	3.30 abc	2.45 bcd	7.11
	KG 30076	5.5 cd	3.9 bcd	2.70 bcd	2.14 cde	8.35
Controls	IAC Caiapó	5.0 cd	5.0 bcd	3.60 ab	2.86 ab	14.30
	IAC 503	18.5 ab	7.4 ab	3.55 ab	2.89 ab	21.39
	V 12549	30.5 a	11.3 a	4.20 a	3.35 a	37.86
Mean		7.0	3.3	2.26	2.03	
F test		8.44**	7.18**	15.27**	23.40**	
CV (%)		15.67	12.89	19.51	14.52	

* Means followed by the same letter in a column did not differ significantly from each other according to Tukey’s test at 5% error probability.

^a^ Means of seven evaluations conducted during plant development. AI = mean number of thrips × mean symptom score.

** Significant at 1% error probability to F’s test.

When averaged over all eight evaluations conducted during the 2012/2013 growing season, accession GKP 10017 (*A*. *cardenasii*) showed the least amount of thrips damage. Among the amphidiploids, we found that An 8 (*A*. *magna x A*. *cardenasii*)^4x^, An 9 (*A*. *gregoryi x A*. *stenosperma*)^4x^ and An 2 (*A*. *gregoryi x A*. *linearifolia*)^4x^ were the least damaged (Table 3).

The amphidiploids with the lowest AI values in the second year were An 8 (*A*. *magna x A*. *cardenasii*)^4x^ and An 9 (*A*. *gregoryi x A*. *stenosperma*)^4x^. The accessions with the best indices were V 7635 (*A*. *vallsii*), GKP 10017 (*A*. *cardenasii*), V 13250 (*A*. *kempff- mercadoi*), K 9484 (*A*. *batizocoi*), Wi 1118 (*A*. *williamsii*), V 14167 (*A*. *duranensis*), and V 13751 (*A*. *magna*) (Table 3). Once again, when compared to their parents, amphidiploids had AI values that were either intermediate, close to that of one parent, or higher than that of both parents. No amphidiploids showed greater resistance than both parents.

**Fig 3:**
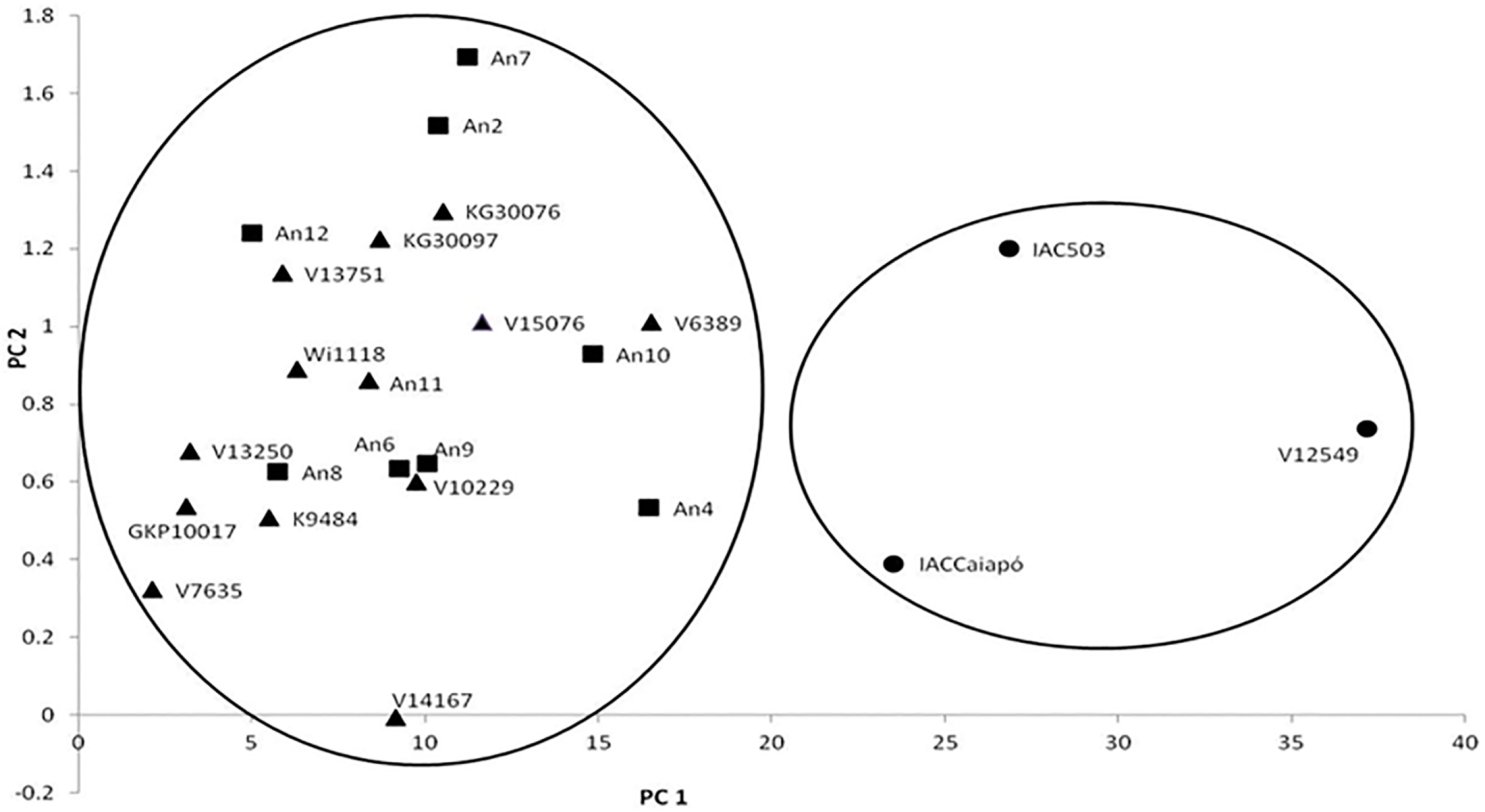
Biplot graph showing grouping of wild species (triangles), amphidiploids (squares) and genotypes of *A*. *hypogaea* (circles) based on principal component analysis of thrips resistance data.

Fig 3 shows the dispersion of the studied genotypes with regard to thrips resistance. The two-season results are based on the average number of thrips per 10 leaflets, the visual symptom score, and the attack index. Principal component analysis revealed that all three variables were correlated. The variable that best explained the dispersion was the attack index, followed by the number of thrips per 10 leaflets. The most susceptible genotypes were grouped (IAC 503, IAC Caiapó, and V 12549), while wild accessions and amphidiploids were dispersed on the left side of the graph. Furthermore, it can be seen that the amphidiploids are not necessarily near the parental accessions, and can be between the two parents or even far away from them on the graph. This shows that resistance to thrips is observed both in diploid species and in diploid and tetraploid synthetic hybrids. It should be noted that the less resistant wild and amphidiploid genotypes (*A*. *gregoryi* V 6389, An 4, and An 10) in the analysis of variance were also closer to the controls (*A*. *hypogaea*). It is noteworthy that the An 4 amphidiploid, which is closest to the grouping of *A*. *hypogaea*, was obtained from the cross between *A*. *ipaënsis* and *A*. *duranensis*, the leading candidates for ancestors of the cultivated peanut [19].

### Morphoagronomic traits

The morphological and reproductive data from the 2011/2012 season are presented in Table 4. Only the amphidiploid An 6 (*A*. *batizocoi x A*. *cardenasii*)^4x^ exhibited a main stem height similar to that of the *A*. *hypogaea* controls. For average branch length and average number of branch nodes, no amphidiploid resembled the controls, whereas for length between nodes, only amphidiploids An 9 (*A*. *gregoryi x A*. *stenosperma*)^4x^ and An 11 (*A*. *vallsii x A*. *stenosperma*)^4x^ had values similar to those of the controls.

**Table 4:** Morphological and reproductive traits of peanut amphidiploids, parents, and cultivars evaluated at Pindorama during 2011/2012 summer season.

Genotypes		Morphological traits*				Reproductive traits*	
		MSH (cm)	ABL (cm)	ABN	LBN (cm)	NSP	MSW (mg)
An 2 -	(V 6389 x V 9401)^4x^	21.6 b	66.3 b	18.8 b	3.93 c	3.2 f	130.0 e
An 4 -	(KG 30076 x V 14167)^4x^	26.7 a	78.1 b	19.3 b	4.54 b	4.7 f	160.9 d
An 6 -	(K 9484 x GKP 10017)^4x^	11.8 d	71.8 b	22.6 a	3.53 c	152.9 b	183.0 d
An 7 -	(V 7635 x Wi 1118)^4x^	22.7 b	61.5 b	18.1 b	3.78 c	4.3 f	188.0 d
An 8 -	(V 13751 x GKP 10017)^4x^	8.1 e	55.4 b	16.6 b	3.95 c	1.9 f	200.6 d
An 9 -	(V 6389 x V 12488)^4x^	10.2 e	63.0 b	19.9 b	3.15 d	99.7 c	189.9 d
An 10 -	(KG 30097 x V 15076)^4x^	19.9 c	63.9 b	17.9 b	3.91 c	9.0 f	207.5 d
An 11 -	(V 7635 x V 10229)^4x^	23.6 b	61.7 b	16.5 b	3.30 d	1.7 f	191.1 d
An 12 -	(K 9484 x V 13250)^4x^	17.8 c	69.0 b	17.3 b	4.25 c	3.0 f	161.0 d
Parents	V 15076	10.2 e	67.1 b	21.2 a	3.33 d	109.9 c	191.1 d
	V 6389	19.9 c	91.8 a	18.6 b	6.00 a	3.8 f	130.0 e
	GKP 10017	5.2 e	64.2 b	22.8 a	3.35 d	59.9 d	68.8 e
	V 13751	9.2 e	75.8 b	18.7 b	4.52 b	38.9 e	110.0 e
	Wi 1118	9.8 e	64.6 b	17.5 b	3.75 c	71.3 d	122.0 e
	V 7635	29.0 a	63.2 b	18.1 b	3.75 c	89.4 c	158.9 d
	K 9484	21.5 b	96.9 a	21.2 a	5.68 a	23.1 e	111.0 e
	V 10229	12.7 d	72.6 b	20.8 b	3.50 c	99.6 c	198.0 d
	KG 30097	11.3 d	58.4 b	18.0 b	3.52 c	46.2 d	184.1 d
	V 14167	13.1 d	65.6 b	22.0 a	3.13 d	68.1 d	113.0 e
	KG 30076	11.1 d	67.4 b	17.9 b	4.00 c	33.6 e	182.9 d
	V 13250	7.6 e	71.8 b	23.9 a	3.75 c	269.4 a	103.0 e
Controls	IAC Caiapó	15.2 d	30.3 c	11.9 c	2.78 d	114.4 c	742.8 c
	V 12549	14.7 d	24.8 c	11.1 c	2.23 d	14.9 e	1180.3 a
	IAC 503	13.5 d	30.0 c	11.4 c	2.80 d	96.3 c	862.9 b
Média		15.1	64.0	18.4	3.8	59.1	252.9
Teste F		22.54**	12.19**	7.04**	6.77**	21.44**	137.23**
CV (%)		17.80	14.93	13.47	17.03	27.64	18.35

* Means followed by the same letter in a column did not differ significantly from each other according to Scott-Knott’s test at 5% error probability.

MSH = main stem height (cm); ABL = average branch length (cm); ABN = average number of branch nodes; LBN = length between nodes (cm); NSP = number of seeds per plant; MSW = mean seed weight (mg).

** Significant at 1% error probability to F’s test.

With regard to reproductive traits, it was found that the accessions of wild species and the amphidiploids were less productive than the *A*. *hypogaea* controls, with the exception of accessions V 13250 (*A*. *kempff-mercadoi*) and V 15076 (*A*. *stenosperma*) and amphidiploids An 6 (*A*. *batizocoi x A*. *cardenasii*) ^4x^ and An 9 (*A*. *gregoryi x A*. *stenosperma*) ^4x^ (Table 4).

In the 2012/2013 season, we chose to evaluate only the amphidiploids (Table 5). Amphidiploids An 8 (*A*. *magna x A*. *cardenasii*)^4x^ and An 6 (*A*. *batizocoi x A*. *cardenasii*)^4x^ had the shortest main stem heights. Number of primary branches and average number of branch nodes (data not shown) did not differ between genotypes. The amphidiploid with the shortest average branch length was An 7 (*A*. *vallsii x A*. *williamsii*) ^4x^ with a value of 61.9 cm, which was nearly twice as long as that of the control genotypes. Reproductive traits followed the same trend observed in the previous season. The number of seeds per plant (NSP) had a high CV because the quantitative behavior of this characteristic.

**Table 5:** Morphological and reproductive traits of the tested amphidiploids evaluated at Pindorama during 2012/2013 summer season.

Genotypes		Morphological traits*				Reproductive traits*	
		MSH (cm)	NPB	ABL (cm)	LBN	NSP	MSW
							(mg)
An 2 -	(V 6389 x V 9401)^4x^	30.4 a	9.0	117.8 a	33.8	23.2 b	137.8 c
An 4 -	(KG 30076 x V 14167)^4x^	36.3 a	11.9	95.6 ab	27.2	21.6 b	186.8 b
An 6 -	(K 9484 x GKP 10017)^4x^	17.8 b	7.9	105.6 ab	30.5	140.3 a	201.0 b
An 7 -	(V 7635 x Wi 1118)^4x^	36.5 a	7.0	61.9 b	24.6	9.6 b	255.5 a
An 8 -	(V 13751 x GKP 10017)^4x^	14.7 b	8.7	88.6 ab	26.5	6.9 b	137.1 c
An 9 -	(V 6389 x V 12488)^4x^	27.4 a	7.7	85.0 ab	29.9	169.5 a	199.5 b
An 11 -	(V 7635 x V 10229)^4x^	36.1 a	9.9	81.8 ab	27.9	10.1 b	265.3 a
Mean		28.5	8.9	90.9	28.6	54.5	197.6
F test		7.26 **	2.63	3.53*	2.30 ns	19.76**	10.86**
CV (%)		23.64	23.02	20.98	14.83	57.32	15.51

* Means followed by the same letter in a column did not differ significantly each other according to Scott-Knott’s test at 5% error probability.

MSH = main stem height (cm); NPB = number of primary branches per plant; ABL = average branch length (cm); LBN = length between nodes (cm); NSP = number of seeds per plant; MSW = mean seed weight (mg).).

** Significant at 1% error probability to F’s test.

## Discussion

Thrips infestations of runner peanut cultivars were most severe between 15 and 54 DAP [7]. Lourenção et al. [5], who evaluated cultivars treated with chemical insecticides to control thrips, reported that regardless of cultivar, the most severe infestation was observed at the third evaluation, conducted at 56 days after seeding. Thereafter, there was a decreasing trend in the number of thrips per leaflet until the end of the plant cycle. In contrast, the most severe infestations of wild species in the current study occurred later, at 85 and 62 DAP in the 2011/2012 and 2012/2013 growing seasons, respectively. Rainfall is one of the factors that can reduce the occurrence of thrips by washing off and drowning individuals and by ensuring moisture levels that are favorable for microorganisms that cause morbidity and mortality in this insect pest [20].

Previous studies of *E*. *flavens* infestation of wild *Arachis* species and peanut cultivars have been conducted with the former also evaluating two synthetic amphidiploids that were included in the present study, An 2 - (*A*. *gregoryi* x *A*. *linearifolia*)^4x^ and An 4 - (*A*. *ipaensis* x *A*. *duranensis*)^4x^ [10, 17]. Both amphidiploids were more resistant to thrips when compared to the evaluated *A*. *hypogaea* genotypes[10]. These authors emphasized the higher incidence of insects in commercial cultivars and suggested that due to their wide genetic variability, wild species are a promising source of resistance gene transfer to the cultivated peanut via synthetic amphidiploids of wild species. Runner IAC 886, IAC Caiapó and IAC 503 were the cultivars with the highest infestation rates and symptom scores. Accessions GKP 10017 (*A*. *cardenasii*) and V 7639 (*A*. *kuhlmannii*) exhibited similar levels of resistance to both *E*. *flavens* and the red-necked peanut worm, *Stegasta bosquella* (Chambers) [17].

In the current study, the amphidiploids An 12 (*A*. *batizocoi x A*. *kempff- mercadoi*) ^4x^, An 8 (*A*. *magna x A*. *cardenasii*) ^4x^ and An 11 (*A*. *vallsii x A*. *stenosperma*) ^4x^ had the lowest attack indexes. Studies have highlighted the importance of wild species as a source of pest resistance [21, 17]; however, this is the first report of thrip resistance in an amphidiploid peanut species.

It is interesting to note that the amphidiploid An 8 stood out in terms of low insect incidence and visual symptom scores over both years of the study, reinforcing the idea that incorporating resistance to *E*. *flavens* (which is known to infest wild species of the genus *Arachis*) can be achieved using synthetic amphidiploids in future crosses with *A*. *hypogaea* breeding lines. It should be noted that there are great differences between the amphidiploids as well as between the amphidiploids and their parents. The evaluation of accessions in terms of resistance to thrips is a good indication of how they will behave when the amphidiploids are obtained. However, assessment of these synthetic hybrids is absolutely essential for the confirmation of their behavior after crossing two different genomes. Thus, in addition to obtaining a large number of amphidiploids, one must select those that are most resistant, which will allow gene pyramiding and more horizontal resistance.

Agronomic characterization is of great importance in studies involving synthetic amphidiploids and accessions of wild species. Although amphidiploids are currently the most feasible route for the introgression of resistance genes from wild species to the cultivated peanut, these amphidiploids closely resemble the wild species in terms of architecture and seed production.

Currently, there are no commercial peanut cultivars that have been genetically modified for pest resistance, partly because the costs associated with regulation and release are extremely high for species considered to be "minor crops," and partly because, to date, the efficiency of genetic techniques for controlling peanut pests is not as high as that for other crops such as corn, soybean, and cotton [22]. Thus, the search for sources of resistance to pests and diseases in wild peanut species remains a useful alternative for reducing the use of pesticides and consequently production costs.

Our findings support the use of amphidiploids in peanut breeding programs. Backcrossing of the best amphidiploids with *A*. *hypogaea* elite lines followed by selection of thrips resistant progeny can potentially lead to the development of new thrips resistant peanut cultivars based on genes found in wild *Arachis* species.

To conclude, accessions of wild *Arachis* species with the lowest levels of *E*. *flavens* infestation over both years of the study were: V7635 (*A*. *vallsii*), V13250 (*A*. *kempff-mercadoi*), and K 9484 (*A*. *batizocoi*). However, other accessions that showed high levels of resistance under the environmental conditions of the 2011/2012 growing season were: Wi1118 (*A*. *williamsii*), V14167 (*A*. *duranensis*) and V13751 (*A*. *magna*). These six accessions are therefore the most promising for the production of new amphidiploids.

The tested amphidiploids with the lowest levels of *E*. *flavens* infestation were An 12 (*A*. *batizocoi x A*. *kempff-mercadoi*)^4x^, An 9 (*A*. *gregoryi x A*. *stenosperma*)^4x^ and An 8 (*A*. *magna x A*. *cardenasii*)^4x^. The latter has high level of resistance to *E*. *flavens* and should therefore be used in future crosses.

Amphidiploids resemble wild species in terms of their architecture and reproductive characteristics.

## Supporting information

S1 Table Raw data of nine amphidiploids and 15 accessions of *Arachis* evaluated during two summer seasons for resistance to thrips and by morphological and reproductive traits. (PDF)
