# Supplementary material for: Resistance to thrips (Enneothrips flavens) in wild and amphidiploid Arachis species
Source: PLoS One. 2017 May 4;12(5):e0176811. doi: 10.1371/journal.pone.0176811 (PMC5417527; doi:10.1371/journal.pone.0176811)
Supplement: S1 Table — (PDF) [file pone.0176811.s001.pdf]

Genotypes	2011/12 Season													
	Number of thrips/10leaflets							Symptom Score						
	35 DAP	45 DAP	55 DAP	65 DAP	75 DAP	85 DAP	95 DAP	35 DAP	45 DAP	55 DAP	65 DAP	75 DAP	85 DAP	95 DAP
An2	0.00	3.33	2.00	14.00	2.00	10.00	8.00	1.50	3.00	3.00	2.00	2.00	3.00	2.00
An2	0.00	0.00	6.00	4.00	0.00	10.00	10.00	1.40	2.00	2.40	2.40	2.00	1.60	1.60
An2	6.00	4.00	6.00	6.00	0.00	14.00	12.00	1.40	2.00	2.00	1.75	1.25	1.75	1.75
An2	10.00	10.00	0.00	2.00	0.00	4.00	2.00	1.60	2.25	2.00	2.50	1.25	1.75	1.50
An4	8.00	4.00	14.00	6.00	2.50	4.00	6.00	2.00	3.33	2.00	3.00	4.00	2.67	3.00
An4	8.00	2.00	12.00	16.00	2.00	4.00	10.00	2.00	3.67	2.33	2.67	2.33	2.67	2.67
An4	6.00	10.00	4.00	2.00	2.00	6.00	0.00	2.20	2.75	3.00	3.25	2.75	2.33	2.33
An4	4.00	4.00	10.00	6.00	4.00	2.00	8.00	1.80	4.00	4.50	4.00	3.50	1.50	3.00
An6	6.00	2.00	10.00	4.00	2.00	2.00	8.00	2.40	3.67	2.67	2.33	3.33	2.33	2.00
An6	4.00	2.00	0.00	4.00	10.00	2.00	0.00	3.40	2.80	2.20	2.00	2.40	2.00	1.60
An6	14.00	4.00	2.00	8.00	2.00	8.00	2.00	2.20	2.75	3.25	3.25	3.00	2.50	1.50
An6	2.00	2.00	4.00	4.00	2.00	6.00	2.00	2.40	3.80	2.80	2.60	2.20	1.60	1.00
An7	12.00	6.00	0.00	22.00	4.00	16.00	6.00	2.20	2.67	1.67	2.00	2.67	2.67	2.33
An7	0.00	12.00	8.00	2.00	0.00	22.00	0.00	1.60	1.50	1.50	1.50	1.00	1.50	2.00
An7	0.00	8.00	2.00	8.00	8.00	10.00	8.00	2.20	1.67	2.33	2.33	2.33	1.67	1.33
An7	2.00	4.00	4.00	0.00	2.00	24.00	10.00	2.60	1.25	2.00	1.75	2.25	1.75	1.40
An8	13.33	0.00	0.00	2.00	2.00	4.00	2.00	2.00	3.00	2.00	1.00	1.50	1.00	1.50
An8	0.00	4.00	6.00	4.00	4.00	2.00	4.00	2.00	2.00	2.25	1.33	2.40	1.80	2.60
An8	0.00	4.00	0.00	0.00	6.00	6.00	6.00	1.80	2.67	2.33	3.00	2.00	2.00	2.00
An8	2.00	4.00	4.00	12.00	0.00	4.00	0.00	1.60	2.33	2.00	2.67	1.67	2.67	2.00
An9	10.00	4.00	2.00	10.00	6.00	4.00	8.00	2.60	3.50	3.50	2.25	2.25	2.00	3.00
An9	8.00	4.00	4.00	12.00	2.00	2.00	10.00	3.20	3.25	3.00	3.00	3.00	2.25	2.33
An9	18.00	2.00	6.00	8.00	0.00	6.00	4.00	2.20	3.33	2.00	2.67	1.67	1.67	2.67
An9	4.00	6.00	8.00	12.00	0.00	2.00	4.00	1.80	2.60	4.20	3.00	2.00	1.20	1.20
An10	2.00	2.00	6.00	10.00	8.00	2.00	6.00	2.80	2.80	2.40	2.80	2.40	2.40	2.60
An10	4.00	2.00	6.00	8.00	2.00	2.00	6.00	1.60	2.80	1.40	2.60	2.40	1.40	2.00
An10	6.00	0.00	0.00	6.00	6.00	2.00	10.00	2.00	3.33	3.33	3.33	2.33	2.33	1.00
An10	6.00	2.00	0.00	10.00	12.00	30.00	2.00	2.40	2.50	3.25	2.75	2.75	2.25	2.75

An11	2.00	0.00	2.00	16.00	0.00	10.00	14.00	2.40	2.80	2.80	2.00	2.00	2.40	1.80
An11	0.00	2.00	10.00	6.00	8.00	4.00	6.00	2.20	3.00	2.60	1.60	1.80	1.75	2.20
An11	0.00	2.00	6.00	4.00	4.00	8.00	2.00	2.20	1.60	1.40	1.60	1.40	1.80	1.80
An11	2.00	4.00	8.00	6.00	2.00	6.00	4.00	2.20	1.50	2.50	1.50	1.25	1.67	2.60
An12	5.00	0.00	4.00	0.00	0.00	0.00	0.00	1.50	2.50	1.00	2.00	1.00	1.00	1.00
An12	0.00	3.33	0.00	0.00	4.00	10.00	2.50	1.00	1.00	1.00	2.00	2.00	1.00	1.00
An12	10.00	2.00	2.50	2.00	2.00	16.00	0.00	1.75	1.00	2.00	3.00	1.00	1.00	1.00
An12	5.00	1.80	2.20	0.70	2.00	8.70	0.80	1.42	1.50	1.33	2.33	1.33	1.00	1.00
GKP10017	0.00	4.00	8.00	2.00	0.00	0.00	2.00	1.80	2.60	1.40	2.80	2.20	1.00	1.20
GKP10017	2.50	3.33	2.00	2.00	0.00	4.00	0.00	1.00	1.50	1.50	2.50	1.50	1.00	1.50
GKP10017	0.00	4.00	2.00	2.00	0.00	4.00	4.00	1.20	1.67	1.67	1.00	1.67	1.67	1.33
GKP10017	4.00	0.00	0.00	4.00	0.00	7.50	2.00	1.00	1.75	1.50	2.00	1.25	1.33	1.00
K9484	2.00	6.00	10.00	4.00	0.00	12.00	0.00	1.20	3.33	1.67	3.00	3.00	1.33	1.50
K9484	2.00	4.00	2.00	6.00	10.00	6.00	0.00	1.60	1.20	1.20	1.25	2.20	1.20	3.00
K9484	4.00	2.00	8.00	4.00	0.00	0.00	0.00	1.20	1.80	2.20	2.60	2.60	1.60	1.20
K9484	2.00	2.00	6.00	2.00	2.00	6.00	0.00	1.80	1.80	2.00	2.60	1.20	1.25	2.25
KG30076	0.00	6.00	2.00	2.00	2.00	2.00	6.00	1.80	2.00	1.40	2.40	1.50	1.40	1.50
KG30076	2.00	6.00	4.00	12.00	0.00	0.00	6.00	1.20	1.60	1.60	2.40	1.80	1.60	2.00
KG30076	20.00	6.00	6.00	10.00	6.00	0.00	6.00	1.40	2.20	2.20	2.40	3.40	2.40	2.00
KG30076	4.00	10.00	6.00	16.00	4.00	6.00	4.00	1.60	2.40	1.80	3.20	2.00	1.00	1.80
KG30097	0.00	2.00	6.00	6.00	0.00	0.00	14.00	1.80	2.80	1.20	2.20	1.40	1.80	2.20
KG30097	6.00	2.00	2.00	16.00	2.00	10.00	4.00	1.60	2.00	1.67	2.33	2.67	2.33	2.33
KG30097	2.00	14.00	16.00	6.00	0.00	4.00	2.00	2.00	1.80	2.00	2.40	1.60	2.40	1.60
KG30097	4.00	2.00	8.00	18.00	4.00	4.00	2.00	1.60	1.80	1.80	2.40	1.80	1.40	1.50
V6389	14.00	6.00	2.00	18.00	6.00	8.00	2.50	3.60	4.00	4.50	3.75	3.50	2.75	3.25
V6389	8.00	4.00	4.00	6.00	6.00	2.00	0.00	2.00	3.75	3.00	1.75	3.50	2.50	2.75
V6389	2.00	8.00	6.00	10.00	2.00	32.00	4.20	1.60	3.00	2.75	4.00	3.25	2.75	2.25
V6389	8.00	8.00	2.00	14.00	12.00	8.00	10.00	2.75	4.00	5.00	4.00	3.00	3.00	2.50
V7635	0.00	2.00	8.00	2.00	2.00	4.00	4.00	1.60	2.00	1.33	1.67	1.00	1.00	1.33
V7635	0.00	0.00	4.00	2.00	0.00	4.00	4.00	1.40	1.67	1.00	1.00	1.00	1.33	1.33
V7635	0.00	2.00	0.00	0.00	0.00	0.00	0.00	1.40	1.00	1.25	1.00	1.00	1.00	1.33

V7635	0.00	0.00	2.00	4.00	0.00	2.00	0.00	1.20	1.50	1.00	1.50	1.00	1.00	1.00
V10229	2.00	4.00	4.00	16.00	2.00	10.00	0.00	2.00	3.67	2.67	2.33	3.67	2.00	1.33
V10229	4.00	2.00	0.00	10.00	12.00	2.00	2.00	2.20	4.00	4.00	3.00	3.67	2.33	3.00
V10229	4.00	8.00	6.00	8.00	2.00	6.00	4.00	1.60	2.67	3.33	2.33	2.67	1.33	2.00
V10229	6.00	0.00	4.00	10.00	4.00	2.00	4.00	2.40	3.00	3.67	2.00	2.33	2.33	1.33
V13250	0.00	4.00	2.00	10.00	0.00	2.00	2.00	1.20	1.00	1.25	1.50	1.00	1.00	1.00
V13250	0.00	2.00	0.00	4.00	0.00	4.00	0.00	1.60	1.75	1.00	2.00	1.50	1.00	1.25
V13250	0.00	4.00	0.00	0.00	4.00	6.00	0.00	1.20	1.25	1.25	1.67	1.00	1.33	1.33
V13250	0.00	0.00	2.00	2.00	0.00	0.00	10.00	1.40	2.00	1.80	1.60	1.40	1.00	1.20
V13751	0.00	2.00	2.00	8.00	2.00	10.00	2.00	1.80	1.75	1.00	1.25	1.25	1.25	1.50
V13751	6.00	4.00	8.00	2.00	6.00	2.00	6.00	1.80	1.25	1.50	1.50	1.25	1.40	1.20
V13751	4.00	2.00	2.00	2.00	8.00	0.00	6.00	1.60	1.67	1.67	1.67	1.33	1.00	1.67
V13751	2.00	14.00	2.00	8.00	0.00	10.00	4.00	1.80	1.75	2.00	1.50	1.00	1.25	2.00
V14167	0.00	0.00	4.00	0.00	8.00	6.00	3.33	2.00	4.00	4.50	4.00	3.50	2.50	3.00
V14167	2.00	4.00	8.00	24.00	0.00	6.00	2.50	1.80	3.67	3.00	3.00	3.67	2.67	2.67
V14167	8.00	2.00	6.00	0.00	2.00	0.00	6.00	1.60	2.50	4.25	3.25	3.50	2.75	1.50
V14167	4.00	4.00	6.00	14.00	0.00	4.00	2.00	1.60	3.00	4.40	3.00	3.00	2.75	2.80
V15076	10.00	2.00	2.00	22.00	6.00	4.00	2.00	2.60	3.33	2.67	3.00	2.67	1.67	1.33
V15076	12.00	4.00	4.00	14.00	8.00	10.00	6.00	1.20	3.67	4.00	2.33	2.67	1.67	2.67
V15076	26.00	2.00	4.00	8.00	0.00	18.00	0.00	1.60	2.50	1.50	3.75	2.50	2.00	2.25
V15076	10.00	2.00	2.00	2.00	2.00	2.00	2.00	1.80	3.00	3.00	2.50	1.50	1.50	1.00
Wi1118	3.33	4.00	2.00	8.00	4.00	8.00	2.50	2.00	2.00	1.75	2.00	2.00	1.33	1.67
Wi1118	0.00	4.00	0.00	2.00	0.00	10.00	2.00	1.40	2.33	2.00	2.67	1.33	1.33	1.00
Wi1118	6.00	12.00	4.00	8.00	2.00	4.00	2.00	1.40	1.25	2.00	2.75	2.50	1.25	1.25
Wi1118	6.00	4.00	0.00	10.00	0.00	4.00	2.00	1.60	2.80	2.80	2.80	1.60	2.50	1.60
Caiapó	28.00	2.00	14.00	8.00	10.00	4.00	10.00	3.80	3.60	2.40	2.40	3.20	2.80	3.60
Caiapó	12.00	6.00	4.00	4.00	12.00	20.00	2.00	3.60	3.50	1.75	3.00	2.75	2.75	3.25
Caiapó	26.00	6.00	6.00	4.00	4.00	20.00	18.00	3.80	3.60	2.40	3.00	2.80	3.60	3.00
Caiapó	14.00	16.00	6.00	6.00	0.00	12.00	6.00	3.40	2.60	3.00	2.40	2.80	2.80	2.80
V12549	22.00	4.00	0.00	26.00	16.00	6.00	12.00	3.60	3.50	1.50	2.25	2.50	3.00	3.50
V12549	16.00	4.00	6.00	12.00	10.00	27.50	24.00	3.60	2.75	1.75	3.50	3.25	3.50	3.50

V12549	36.00	6.00	4.00	2.00	8.00	20.00	28.00	4.00	3.00	2.33	3.00	2.00	3.67	3.00
V12549	14.00	2.00	4.00	0.00	2.00	10.00	5.00	2.50	1.50	2.50	1.50	2.50	2.00	3.00
IAC 503	14.00	2.00	8.00	16.00	10.00	14.00	20.00	3.80	2.20	2.40	2.40	2.80	3.00	2.20
IAC 503	14.00	12.00	6.00	2.00	6.00	27.50	8.00	3.20	3.20	1.80	2.60	3.00	2.00	2.60
IAC 503	4.00	2.00	2.00	0.00	0.00	4.00	12.00	3.00	2.80	2.00	2.40	1.60	2.40	3.20
IAC 503	6.00	2.00	2.00	6.00	10.00	92.00	5.00	3.60	3.60	2.40	2.40	2.00	2.60	3.20

*fields in blank - genotypes not evaluated

MSH = main stem height (cm); ABL = average branch length (cm); ABN = average number of branch nodes; LBN = length between nodes (cm); NSP = n

MSH	ABL	LBN	ABN	NSP	MSW	Number of thrips/10leaflets									
						45 DAP	52 DAP	62 DAP	70 DAP	77 DAP	84 DAP	98 DAP	110 DAP	45 DAP	52 DAP
28.00	84.00	3.00	22.00	7.2	134.7	2.5	2.0	6.0	0.0	0.0	6.0	4.0	2.0	1.3	1.8
17.80	68.60	4.20	18.40	2.5	140.0	2.0	4.0	4.0	2.0	0.0	12.0	0.0	0.0	1.8	1.8
23.67	57.50	4.00	20.25	3.2	115.6	10.0	0.0	4.0	2.0	0.0	2.0	6.0	0.0	1.0	1.0
16.75	55.25	4.50	14.50	0.0		20.0	10.0	18.0	0.0	18.0	0.0	2.0	8.0	1.7	1.3
24.33	45.67	3.67	12.00	3.0	166.7	2.0	4.0	18.0	2.0	2.0	14.0	2.0	2.0	2.4	2.0
31.00	78.00	4.33	22.00	6.0	166.7	7.5	5.0	4.0	0.0	0.0	8.0	0.0	36.0	2.5	2.0
31.33	79.33	4.67	21.00	4.0	147.5	3.3	0.0	6.0	0.0	4.0	0.0	0.0	4.0	1.3	1.0
20.00	85.00	5.50	18.00	5.6	162.5	0.0	0.0	14.0	0.0	10.0	4.0	0.0	16.0	4.5	2.0
13.67	72.67	3.33	24.00	125.0	160.0	0.0	0.0	4.0	4.0	2.0	4.0	0.0	2.0	2.5	2.0
12.60	70.40	4.40	20.20	206.0	160.7	0.0	0.0	0.0	4.0	0.0	4.0	0.0	2.0	1.7	2.3
9.75	66.00	3.00	23.75	125.7	237.1	3.3	0.0	12.0	0.0	2.0	4.0	0.0	6.0	2.3	2.0
11.00	78.20	3.40	22.40	155.0	174.2	30.0	0.0	8.0	2.0	0.0	4.0	2.0	0.0	2.5	1.0
22.33	53.67	3.33	16.33	1.7	216.0	0.0	0.0	10.0	2.0	0.0	0.0	2.0	2.0	1.5	1.8
26.00	66.00	4.50	17.50	3.0	136.7	0.0	15.0	0.0	2.0	0.0	0.0	0.0	6.0	2.5	1.5
17.67	52.33	3.00	18.67	5.5	174.5	5.0	0.0	2.0	2.0	10.0	0.0	0.0	4.0	2.5	1.5
24.75	74.00	4.25	20.00	7.2	225.0	3.3	16.7	2.0	6.0	2.0	2.0	6.0	5.0	1.3	1.0
10.00	47.50	4.50	11.00	2.5	220.0	2.0	0.0	4.0	0.0	0.0	0.0	0.0	2.0	2.0	1.4
7.20	50.60	3.00	19.20	1.7	147.1	0.0	0.0	2.0	0.0	0.0	0.0	0.0	0.0	2.0	1.5
8.00	57.67	4.00	17.00	1.5	200.0	10.0	2.5	2.0	2.0	0.0	4.0	2.0	4.0	1.3	1.0
7.00	65.67	4.33	19.00	2.0	175.0	0.0	0.0	6.0	0.0	4.0	6.0	0.0	0.0	2.5	1.3
9.75	59.25	3.50	19.25	80.3	165.1	2.0	0.0	4.0	0.0	0.0	6.0	0.0	0.0	1.2	1.8
11.00	62.50	3.75	19.50	59.3	211.0	0.0	0.0	2.0	0.0	0.0	4.0	0.0	0.0	1.3	1.3
9.67	61.33	2.33	21.67	115.0	210.9	0.0	0.0	0.0	2.0	0.0	2.0	2.0	2.0	1.0	1.0
10.25	69.00	3.00	19.20	144.3	172.6	0.0	10.0	12.0	0.0	2.0	4.0	2.0	2.0	2.0	1.5
15.40	63.00	3.60	16.40	8.2	212.6										
15.60	59.80	3.20	18.80	11.6	188.8										
19.67	72.33	4.33	20.67	10.5	201.9										
17.00	60.25	4.50	15.75	8.8	227.4										

24.00	56.60	2.20	12.40	0.0		5.0	0.0	10.0	2.0	0.0	6.0	0.0	0.0	2.0	1.5
23.20	56.00	3.60	16.80	1.2	200.0	0.0	5.0	2.0	2.0	0.0	0.0	2.0	2.0	1.8	2.8
22.40	66.60	3.60	18.60	3.6	222.2	0.0	0.0	14.0	4.0	0.0	0.0	0.0	6.0	4.0	3.0
24.75	67.75	3.75	18.25	2.1	151.2	7.5	2.5	2.0	0.0	4.0	2.0	2.0	0.0	2.3	1.5
15.00	63.00	4.00	12.00	1.0	160.0	2.0	2.0	4.0	0.0	0.0	4.0	0.0	6.0	1.6	1.8
20.00	48.00	3.00	16.00	0.0		0.0	3.3	6.0	2.0	4.0	0.0	12.0	16.0	2.0	1.3
18.00	96.00	6.00	24.00	9.0	162.2	0.0	0.0	0.0	4.0	2.0	8.0	2.0	4.0	2.0	1.3
						1.0	2.0	3.0	2.0	2.0	4.0	5.0	9.0	1.9	1.5
6.80	68.60	3.40	20.60	27.8	66.5	0.0	0.0	8.0	0.0	0.0	0.0	0.0	0.0	1.3	1.0
3.50	53.00	4.00	23.50	43.0	69.8	0.0	0.0	4.0	0.0	0.0	0.0	0.0	4.0	1.3	1.3
4.33	74.33	3.00	27.00	108.3	67.9	0.0	5.0	2.0	2.0	0.0	0.0	0.0	2.0	1.0	1.8
6.33	60.75	3.00	20.00	60.5	71.1	0.0	0.0	6.0	6.0	0.0	0.0	0.0	0.0	1.0	1.0
20.33	99.33	6.67	21.33	24.5	89.8	0.0	0.0	2.0	2.0	0.0	2.0	2.0	2.0	2.0	1.5
22.80	102.20	5.60	21.00	29.0	137.2	0.0	4.0	0.0	2.0	0.0	0.0	0.0	2.0	3.4	1.6
24.40	99.20	4.80	24.00	22.0	93.6	10.0	0.0	0.0	0.0	0.0	6.0	2.0	4.0	2.7	1.3
18.40	89.00	5.60	18.60	17.0	123.5	0.0	0.0	0.0	0.0	0.0	0.0	0.0	4.0	3.5	1.5
10.75	46.40	3.60	12.80	15.1	145.0	30.0	7.5	8.0	6.0	0.0	2.0	2.0	2.0	3.0	3.3
12.60	71.60	3.40	21.60	30.7	189.8	0.0	0.0	0.0	0.0	4.0	0.0	0.0	12.0	1.3	1.7
11.00	70.00	4.00	19.20	42.0	207.6	0.0	0.0	4.0	2.0	2.0	2.0	2.0	0.0	3.3	1.3
10.00	81.60	5.00	18.00	47.0	189.3	7.5	7.5	10.0	2.0	0.0	2.0	0.0	2.0	2.3	1.3
13.40	60.00	2.60	19.20	41.2	243.8	0.0	0.0	6.0	0.0	2.0	0.0	0.0	10.0	1.0	2.0
10.67	59.67	4.67	14.67	79.7	223.9	0.0	5.0	4.0	0.0	0.0	0.0	0.0	4.0	1.5	1.5
9.40	51.00	3.00	20.00	32.8	252.3	2.0	6.0	4.0	2.0	0.0	4.0	2.0	0.0	1.6	1.4
11.80	62.80	3.80	18.00	43.0	235.0	10.0	0.0	8.0	0.0	0.0	0.0	0.0	4.0	3.0	1.5
19.50	94.50	5.75	19.50	8.8	133.7	0.0	0.0	6.0	0.0	2.0	2.0	2.0	2.0	1.8	1.8
21.00	95.75	6.00	16.50	4.0	150.6	5.0	0.0	6.0	4.0	0.0	6.0	0.0	2.0	4.0	3.5
22.00	125.00	6.25	20.25	9.8	135.1	5.0	2.5	2.0	0.0	0.0	4.0	0.0	12.0	3.8	2.3
17.00	77.00	6.00	13.00	10.5	123.3	0.0	0.0	10.0	0.0	2.0	4.0	0.0	8.0	2.7	1.3
29.67	61.00	3.67	*	85.6	199.6	0.0	0.0	4.0	0.0	0.0	2.0	4.0	0.0	2.0	2.0
33.33	71.00	3.67	16.33	121.0	506.3	0.0	0.0	0.0	0.0	2.0	0.0	0.0	0.0	1.0	1.0
22.00	62.33	3.67	20.33	96.3	302.6	5.0	5.0	2.0	0.0	0.0	0.0	2.0	0.0	2.0	1.5

31.00	58.50	4.00	17.50	54.5	273.8	0.0	0.0	2.0	0.0	0.0	0.0	0.0	0.0	2.0	1.0
14.33	52.33	3.00	15.00	49.7	252.6	0.0	0.0	16.0	0.0	0.0	14.0	2.0	2.0	2.0	1.5
12.67	60.00	3.00	18.33	80.7	175.0	0.0	0.0	0.0	0.0	0.0	4.0	0.0	6.0	1.5	1.0
13.00	88.00	5.00	24.33	69.0	188.4	6.7	3.3	4.0	0.0	0.0	2.0	0.0	4.0	2.0	1.7
10.67	90.00	3.00	25.33	199.0	175.9	2.5	0.0	10.0	2.0	2.0	0.0	0.0	0.0	2.8	1.3
7.67	73.50	3.50	25.00	159.5	147.5	0.0	0.0	2.0	2.0	0.0	4.0	0.0	0.0	1.6	1.6
9.67	73.75	3.25	22.00	350.0	85.7	15.0	0.0	0.0	0.0	0.0	0.0	0.0	2.0	1.5	1.5
6.67	73.00	4.00	23.33	310.0	89.7	0.0	5.0	4.0	2.0	0.0	2.0	2.0	2.0	1.5	1.0
6.20	67.00	4.20	25.40	258.0	89.1	0.0	0.0	6.0	0.0	0.0	2.0	0.0	4.0	2.0	1.0
9.25	79.00	3.75	20.50	39.8	120.4	0.0	0.0	10.0	0.0	0.0	0.0	2.0	2.0	1.8	1.3
10.00	68.50	5.00	17.25	39.3	94.3	4.0	2.0	18.0	0.0	2.0	0.0	0.0	4.0	2.6	2.6
7.67	77.67	4.33	19.67	45.0	115.6	0.0	0.0	6.0	0.0	0.0	0.0	0.0	0.0	1.2	1.6
10.00	78.00	5.00	17.25	31.7	109.8	0.0	0.0	0.0	0.0	0.0	4.0	0.0	0.0	1.5	1.5
9.00	64.50	2.50	23.50	53.5	119.6	0.0	0.0	2.0	2.0	0.0	2.0	0.0	0.0	2.0	1.8
16.67	86.00	3.67	22.67	94.0	112.8	5.0	5.0	16.0	0.0	0.0	8.0	0.0	2.0	1.5	2.0
12.25	54.50	3.25	22.75	66.0	134.8	0.0	0.0	4.0	0.0	0.0	2.0	0.0	0.0	3.0	2.5
14.40	57.40	3.00	19.20	59.0	84.7	0.0	0.0	6.0	0.0	4.0	0.0	2.0	1.0	1.0	1.0
10.33	71.33	3.00	22.67	51.0	190.8	5.0	0.0	24.0	2.0	0.0	2.0	0.0	2.0	2.0	1.5
11.33	57.00	3.00	24.00	74.3	191.4	10.0	0.0	6.0	0.0	4.0	14.0	0.0	2.0	4.0	2.0
11.75	66.00	3.75	18.75	151.0	211.9	0.0	0.0	15.0	1.0	2.0	8.0	0.0	3.0	1.0	1.0
7.50	74.00	3.50	19.50	163.2	170.3	5.0	0.0	15.0	1.0	2.0	8.0	0.0	1.0	*	*
9.00	47.33	3.00	12.33	52.5	133.3	0.0	0.0	6.0	0.0	2.0	0.0	0.0	0.0	1.0	1.0
8.67	64.67	3.67	18.67	64.0	140.2	0.0	0.0	4.0	2.0	2.0	2.0	0.0	2.0	1.4	1.4
10.75	74.00	4.25	21.25	111.0	117.1	0.0	0.0	4.0	2.0	2.0	0.0	2.0	2.0	2.0	1.0
10.60	72.20	4.00	17.80	57.5	97.4	6.0	2.0	2.0	0.0	0.0	6.0	10.0	2.0	1.6	1.4
15.20	29.80	2.80	10.00	120.5	664.0	0.0	4.0	6.0	6.0	4.0	12.0	4.0	6.0	3.8	3.2
12.25	22.00	3.50	10.75	75.0	835.0	2.0	4.0	0.0	4.0	0.0	4.0	4.0	2.0	2.4	2.4
15.80	33.80	2.00	14.20	92.0	760.9	0.0	8.0	10.0	4.0	4.0	32.0	0.0	0.0	4.0	2.0
17.40	35.40	2.80	12.80	170.0	711.8	0.0	6.7	4.0	0.0	4.0	16.0	4.0	0.0	3.3	2.0
19.50	22.00	2.00	9.25	20.7	1325.6	2.0	2.0	28.0	4.0	6.0	14.0	2.0	0.0	3.4	3.4
12.25	26.50	3.00	12.25	15.8	1114.3	20.0	0.0	6.0	2.0	2.0	8.0	2.0	8.0	4.0	3.0

13.00	25.33	2.00	11.33	12.8	1086.3	20.0	13.3	26.0	4.0	12.0	2.0	22.0	6.0	4.3	3.3
14.00	25.50	2.00	11.50	10.3	1195.1	20.0	0.0	42.0	24.0	2.0	14.0	2.0	12.0	5.0	1.0
12.60	27.20	2.20	12.40	90.0	866.7	16.7	16.7	26.0	2.0	4.0	12.0	2.0	10.0	2.7	2.7
12.00	28.60	3.60	10.40	90.0	957.1	0.0	2.0	8.0	16.0	8.0	8.0	2.0	6.0	2.2	2.6
12.00	29.20	2.40	10.40	118.0	825.8	5.0	0.0	14.0	4.0	6.0	2.0	0.0	6.0	2.5	1.5
17.40	34.80	3.00	12.20	87.3	801.8	0.0	7.5	16.0	2.0	0.0	8.0	0.0	8.0	2.8	2.3

umber of seeds per plant; MSW = mean seed weight (mg).

12/13 Season										
Symptom Score						MSH	ABL	ABN	NSP	MSW
62 DAP	70 DAP	77 DAP	84 DAP	98 DAP	110 DAP					
1.2	3	2.8	2.2	1.6	1.2	31.25	119.25	34.50	21.5	148.0
1.6	3	2.6	1.6	2.8	2.2	32.50	111.40	30.00	27.2	126.1
1.4	1.2	1.6	1.8	1.8	1.4	31.00	145.00	39.00	21.0	134.3
1.6	2.4	2.4	1.8	2.4	1.8	27.00	95.50	31.50	23.2	142.7
2.4	2.4	3.4	2.8	2	2.2	25.50	75.75	24.75	16.5	210.0
1.6	3.4	2	2.6	2.2	2.6	36.33	101.00	28.67	27.3	154.4
1.6	2	3.6	1.6	2.2	2.4	29.33	87.67	26.33	7.7	196.1
2.4	4.4	4.6	2.8	2.6	3	54.00	118.00	29.00	35.0	186.8
2	2.4	1.8	1.8	1.8	1.8	19.00	137.00	34.00	210.0	232.0
1.6	2.8	1.6	1.6	2	2.2	21.50	70.00	23.50	93.0	194.9
1.2	2.2	1.2	1.8	2.4	1.2	15.67	98.33	31.00	171.0	197.2
1.6	1.8	1.8	1.4	1.8	2	15.00	117.00	33.50	87.0	179.7
1.6	2.2	3.4	2.2	2	1.4	32.67	50.67	19.33	8.0	255.5
1.6	2	2.4	2.4	2.4	2.2	38.00	73.50	25.50	6.0	288.3
1.4	1.6	1.8	1.6	2.2	1.6	40.50	87.50	32.50	15.0	222.7
1.6	1.8	1.6	2.2	2	1.8	35.00	36.00	21.00	9.5	255.5
1.6	1.4	2	1.6	1.4	1.2	13.50	76.50	18.50	7.0	120.4
1.4	1.6	1.4	1.8	2.2	1.6	14.50	88.00	25.50	6.9	119.7
1.6	1	1.8	1.4	2.4	1.8	15.75	96.75	34.00	5.0	210.5
1.4	1	1.4	1.4	2.2	1.6	15.00	93.00	28.00	8.7	97.7
1.8	2	2.2	1.8	1.8	1.4	17.75	87.25	29.25	191.3	216.3
1.8	1.8	1.8	1.4	1.4	1.2	18.33	82.67	29.00	217.3	189.9
1.6	2	1.8	1.6	2	1.4	39.00	73.00	26.00	100.0	173.7
1.6	4	3.6	2	2	2.2	34.50	97.00	35.50	169.5	218.0

1.2	1.6	2.2	2.4	2	1.6	38.50	81.00	27.00	12.0	227.1
1.8	2	2.6	2.2	2.4	1.6	33.00	73.00	23.00	4.5	297.8
1.4	3	2.6	2	2.4	1.6	36.50	84.75	31.25	18.5	259.9
2	1.4	2	2.2	2.6	2	36.33	88.50	30.50	5.5	276.4
1.6	2.2	4	2.2	2.4	1.6					
1.8	2	1.8	2.2	2.2	1.2					
1.6	2	1.8	2	2.4	2.8					
1.7	2.1	2.5	2.1	2.3	1.9					
1.4	1.2	1.2	1	1.6	1.4					
1.6	1	1	1.6	1.6	1.4					
1.4	1	1.2	1.4	1.8	1.2					
1.2	1.4	1.2	1.2	2	1.2					
1.4	1.6	1	1.6	1.8	2					
1.4	1.6	1.4	2.4	2	2					
1.8	1.8	1	2.2	2.2	3					
2	1.2	3	2.8	2.2	1.6					
1.4	2	2.8	1.8	2	2.2					
1.4	2	3.2	2.4	1.8	1.4					
2	2.2	2.2	3.2	2.4	1.6					
1.4	2	2.6	2	2.2	2					
1.4	2.2	1.6	1.8	1.8	1.6					
1.4	1.6	2.2	1.4	2.4	2					
1.6	1.8	1.8	2	2.4	1.2					
1.2	2.4	4.6	1.6	2.4	1.6					
2	2.8	3.6	2.4	2.6	2.2					
1.8	2.8	3.2	2.6	1.8	1.8					
2.6	1	2.6	1.6	2	2.6					
1.8	1.6	3.8	1.6	2.6	3					
1.4	2.2	1.2	1.4	2.2	1.4					
1.4	1.2	1.2	1.4	1.8	1.8					
1.4	1.6	3.4	1.4	2	1.2					

1.2	1	1.2	1.4	1.4	1.6
2.6	1.8	1.4	2.4	1.8	1.6
1.4	1.8	1.4	1.6	2	1.8
1.8	2.6	1.4	2	2.4	1.2
1.6	1.6	2	1.4	2.4	1.4
1.4	1.4	1.6	1.4	1.8	1.2
1.6	1.4	1.4	1.2	1.2	1.2
1.2	1.2	1.2	1.2	1.2	1.2
1.4	1.6	2	1.4	1.6	1.4
2.4	1.2	1.8	2.2	2	2
1.6	2.4	1.8	1.4	2	3
1.8	2.2	2	1.4	1.6	1
1.2	1.2	2.4	1.8	2	1.6
1.6	2	2.2	1.4	2	2.6
1.8	3	2	2	2	2.4
2	1.4	1.6	1.6	2.2	1.2
1.4	2.4	1.6	2.2	2.4	1.2
1.2	1.6	2.4	1.6	1.6	2.2
1.8	3	2.2	2.2	2.2	2
1.5	2.3	2.3	1.9	1.9	2.4
*	*	*	*	*	*
1.2	1.6	1.2	1.8	1.8	1.2
1.8	2.6	1.8	1.8	2.2	1.2
1.25	1.2	2	2.2	2	1.6
1.4	1.8	2	1.8	2.6	1.2
2	2.6	2.8	2.2	1.8	3
1.6	2.8	3.4	4.2	2.8	3
2.6	3.8	4.2	2	2.8	2.4
2.2	3.4	4	2.6	3.2	2.2
2.4	2.6	4.6	1.8	2.6	3.8
2.8	4.2	4.4	2.8	3.4	2

2.8	3.8	3.8	3.8	3.2	3.6
3	4	4	3.4	3	3
2.6	3	4.2	3	2.2	2.8
2.2	3.6	3	3.6	2.6	2.8
2.8	4	3.4	3.8	2.6	2.6
2.4	3.6	3.6	2.8	2.6	2.8
